# Structural comparisons of host and African swine fever virus dUTPases reveal new clues for inhibitor development

**DOI:** 10.1074/jbc.RA120.014005

**Published:** 2020-11-23

**Authors:** Rui Liang, Gang Wang, Ding Zhang, Gang Ye, Mengxia Li, Yuejun Shi, Jiale Shi, Huanchun Chen, Guiqing Peng

**Affiliations:** 1 State Key Laboratory of Agricultural Microbiology, College of Veterinary Medicine, Huazhong Agricultural University, Wuhan, Hubei Province, China; 2 Key Laboratory of Preventive Veterinary Medicine in Hubei Province, The Cooperative Innovation Center for Sustainable Pig Production, Wuhan, Hubei Province, China; 3 College of Life Science and Technology, Huazhong Agricultural University, Wuhan, Hubei Province, China

**Keywords:** ASFV, swine, dUTPase, variable conformations, inhibitor design, aDUT, ASFV dUTPase, dUMP, 2′-deoxyuridine-5′-monophosphate, dUPNPP, α,β-imido-deoxyuridine triphosphate, PDB, Protein Data Bank, PPi, pyrophosphate, RMSD, root-mean-square deviation, sDUT, swine dUTPase

## Abstract

African swine fever, caused by the African swine fever virus (ASFV), is among the most significant swine diseases. There are currently no effective treatments against ASFV. ASFV contains a gene encoding a dUTPase (E165R), which is required for viral replication in swine macrophages, making it an attractive target for inhibitor development. However, the full structural details of the ASFV dUTPase and those of the comparable swine enzyme are not available, limiting further insights. Herein, we determine the crystal structures of ASFV dUTPase and swine dUTPase in both their ligand-free and ligand-bound forms. We observe that the swine enzyme employs a classical dUTPase architecture made up of three-subunit active sites, whereas the ASFV enzyme employs a novel two-subunit active site. We then performed a comparative analysis of all dUTPase structures uploaded in the Protein Data Bank (PDB), which showed classical and non-classical types were mainly determined by the C-terminal β-strand orientation, and the difference was mainly related to the four amino acids behind motif IV. Thus, our study not only explains the reason for the structural diversity of dUTPase but also reveals how to predict dUTPase type, which may have implications for the dUTPase family. Finally, we tested two dUTPase inhibitors developed for the *Plasmodium falciparum* dUTPase against the swine and ASFV enzymes. One of these compounds inhibited the ASFV dUTPase at low micromolar concentrations (*K*_*d*_ = 15.6 μM) and with some selectivity (∼2x) over swine dUTPase. In conclusion, our study expands our understanding of the dUTPase family and may aid in the development of specific ASFV inhibitors.

African swine fever is an acute and highly infectious disease caused by the African swine fever virus (ASFV). ASFV, the only member of the family Asfarviridae and the only known DNA arbovirus (1), has a full length of 170 to 190 kb and contains 150 to 167 open reading frames (1, 2). ASFV encodes many proteins involved not only in viral particle assembly but also in DNA replication and repair. Furthermore, the ASFV genome encodes a number of proteins involved in the evasion of host defense (2, 3, 4).

African swine fever was first found in Kenya in 1921, after which ASFV rapidly spread through Africa and then to Europe and other locations (2, 5). The virus can be transmitted through direct contact with infected swine or their products or by the bite of soft-bodied ticks of the genus Ornithodoros (1, 2, 6). The disease spreads rapidly among pigs and has a high mortality rate. Although the clinical symptoms of African swine fever are very similar to those of classical swine fever, the two diseases can be distinguished by laboratory diagnostic technology (7). ASFV was transmitted to China in August 2018, resulting in enormous economic losses to the pig industry (1, 5). At present, there are no effective vaccines or drugs for the prevention or treatment of ASFV (8).

Owing to the involvement of DNA in many chemical reactions, numerous mutagenic and carcinogenic modifications occur in genomes (9, 10). Therefore, maintaining DNA integrity is of great importance. Deoxyuridine 5'-triphosphate nucleotidohydrolase (dUTPase, EC 3.6.1.23) is a ubiquitous enzyme in DNA repair that plays a key role in preventing uracil from being incorporated into DNA. dUTPases are widespread in organisms, including eukaryotes, prokaryotes, and some viruses (11, 12, 13). This type of enzyme has dual functions. On the one hand, it catalyzes the cleavage of dUTP into dUMP and pyrophosphate (PPi), which reduces the possibility that dUTP will be incorporated into DNA through DNA polymerase (9). On the other hand, dUTPase produces dUMP, a precursor of dTTP, which contributes to thymidylate biosynthesis and the strict control of cellular dUTP/dTTP ratios. dUTPase deficiency leads to the incorporation of a high level of dUMP into genes. When organisms lack dUTPase, although genes containing uracil activate uracil excision repair, uracil is reincorporated into genes after repair synthesis at a high dUTP/dTTP ratio. Uracil excision repair thus becomes an overactive and futile cycle that eventually leads to cell death through double-stranded DNA breakage (thymine-free cell death) (9, 11).

The potential role of dUTPase antagonism in thymine-free cell death has prompted investigations into the enzymatic mechanism of dUTPase and, in particular, studies of novel anticancer and antimicrobial targets (14, 15). Some drugs, such as fluorouracil or methotrexate, disrupt the cellular dUTP/dTTP ratio, resulting in the synthesis of highly uracil-substituted DNA. Moreover, dUTPase inhibitors can fight infectious diseases such as malaria and tuberculosis (9). It has been reported that dUTPase is closely related to the virulence and highly efficient replication of viruses (16). In addition, the results of recent research suggest that pseudorabies virus dUTPase contributes to immune evasion (17). At present, dUTPases from different species have been widely studied. A striking feature is that the trimeric dUTPase contains five conserved motifs that are involved in the assembly of each of its three active sites (18). In a three-subunit active site, one subunit provides motifs I, II, and IV; another provides motif III; and the third subunit provides the C-terminal P-loop–like motif V. Motif V is rather flexible in the absence of substrate and undergoes a disorder-to-order transition upon ligand binding, thus capping the active site (19, 20). Previous studies have revealed that motif V is not required for substrate binding but is essential for catalysis by contributing several key residues, such as a “Phe-lid” and an arginine finger (10, 18). In addition, the conserved amino acids from motif V also form an interacting network within the active site that is also important for catalysis (21).

Previous research has shown that dUTPase is present in actively dividing and differentiating cells. ASFV mainly infects swine macrophages, which are quiescent cells, so there is no dUTPase in swine macrophages. Therefore, to ensure normal replication in porcine macrophages, ASFV must rely on its own encoded dUTPase (E165R); thus, ASFV dUTPase is considered to be a potential drug target (13, 16, 22). Although the structure of ASFV dUTPase was reported recently (13, 22), the structures belong to the postreaction state, the structure of the C terminus (motif V) has not been analyzed, and the catalytic mechanism has not been elaborated in detail. Moreover, the crystal structure of swine dUTPase has not yet been reported.

In the present work, we obtained three types of ASFV dUTPase (aDUT) structures by X-ray crystallography, namely, a ligand-free aDUT, an aDUT-dUPNPP-Mg^2+^ complex (prereaction state), and an aDUT-dUMP complex (postreaction state). A nonclassical two-subunit active site was proposed by comparing the pre- and postreaction states, and key amino acid sites were verified by mutant enzyme activity experiments. Furthermore, we obtained the crystal structures of swine dUTPase (sDUT) and the sDUT-dUPNPP-Mg^2+^ complex (prereaction state). By comparing the overall structures of ASFV dUTPase and swine dUTPase, our study revealed how to predict whether a dUTPase is a classical or nonclassical dUTPase based on its primary sequence, which will have broader implications for the dUTPase family. Moreover, inhibition studies by enzyme kinetics and surface plasmon resonance (SPR) analyses suggested that inhibitor compound 1 acts as a competitive inhibitor with a *K*_*d*_ of 15.6 μM against ASFV dUTPase. Interestingly, inhibitory activity of compound 1 is selective for ASFV dUTPase over swine dUTPase. In conclusion, we provided valuable structural information that will greatly facilitate the development of targeted inhibitors or specific drugs against ASFV without affecting swine.

## Results

### Overall structures of ASFV dUTPase and swine dUTPase

**Figure 1:**
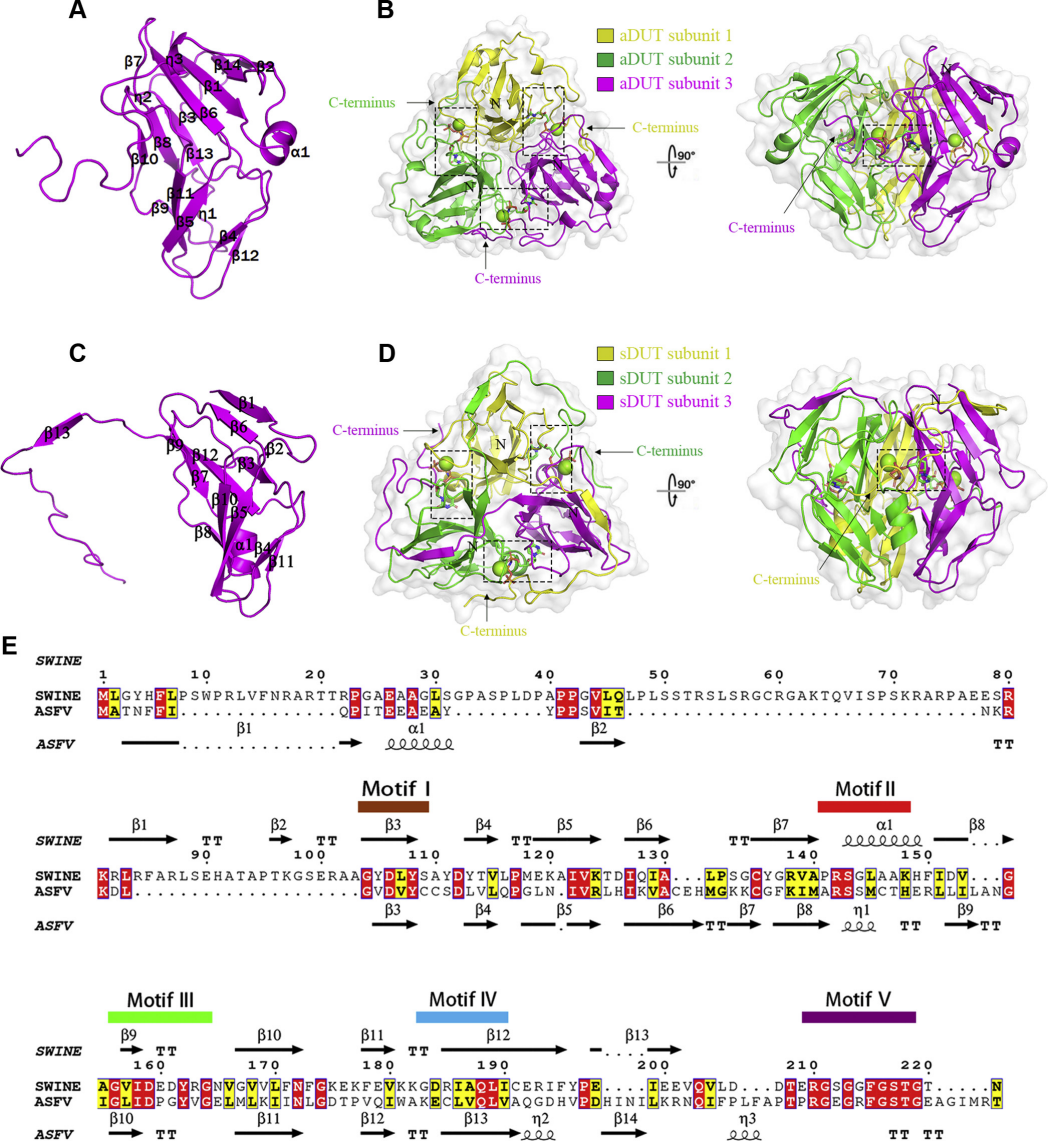
**Overall structures of ASFV dUTPase (aDUT) and swine dUTPase (sDUT).***A*, the secondary structure of one aDUT subunit is presented (magenta). *B*, top view (*left*) and side view (*right*) of aDUT-dUPNPP-Mg^2+^. Different subunits are indicated by different colors. The *black dotted boxes* indicate the substrate-binding sites. The dUPNPP is indicated as *sticks* and colored by element. The magnesium ion is indicated by the *green sphere*. *C*, the secondary structure of one sDUT subunit is shown (*magenta*). *D*, top view (*left*) and side view (*right*) of sDUT-dUPNPP-Mg^2+^. *E*, sequence alignment between ASFV dUTPase and swine dUTPase.

ASFV dUTPase (aDUT) gene consists of 495 bp and encodes 165 amino acid residues. The results of gel filtration experiments on the recombinant protein and static light scattering measurements indicated that ASFV dUTPase is in a trimeric conformation, which is consistent with the results of previous studies (13, 22). Ligand-free ASFV dUTPase crystals grew in the I2_1_3 space group with a subunit in each asymmetric unit, and the structure contained 146 visible residues (residues 1–146); the remaining C-terminal residues (residues 147–165) are flexible. The α,β-bridging oxygen in dUTP was replaced by an imido group to form 2′-deoxyuridine 5′-(α,β-imido) triphosphate (dUPNPP), a nonhydrolyzable substrate analog and potent competitive inhibitor of dUTPase (23). The crystal of the aDUT-dUPNPP-Mg^2+^ complex grew in the P2_1_2_1_2_1_ space group with one trimer in each asymmetric unit (Table 1). Fortunately, clear electron density was observed for the remaining C-terminal residues in the aDUT-dUPNPP-Mg^2+^ complex structure. The full-length subunit and secondary structure distribution of ASFV dUTPase are clearly shown (Fig. 1*A*); in general, residues Ala^2^-Ala^123^ form a β-barrel core and β3-β12 form a stable β-barrel, with α1, β1, β2, β13, β14, and η1 capping the barrel. The three active sites of ASFV dUTPase were found to lie at the interface between the three subunits (Fig. 1*B*).

**Table 1:** Crystallography statistics

	ASFV dUTPase	aDUT-dUPNPP-Mg^2+^	aDUT-dUMP	sDUT-dUPNPP-Mg^2+^
Data collection statistics				
X-ray source	SSRF BL17U1 BL19U1			
Wavelength (Å)	0.97918	0.97853	0.97835	0.97918
Space group	I2_1_3	P2_1_2_1_2_1_	P2_1_2_1_2_1_	F4_1_32
Cell constants a, b, c, α, β, γ	98.01 Å	68.04Å	96.18 Å	162.39 Å
	98.01 Å	68.43 Å	103.06 Å	162.39 Å
	98.01 Å	117.97 Å	102.89 Å	162.39 Å
	90.00°	90.00°	90.00°	90.00°
	90.00°	90.00°	90.00°	90.00°
	90.00°	90.00°	90.00°	90.00°
Resolution (Å)	50.00–2.28	50.00–1.96	50.00–2.0	50.00–1.89
	(2.32–2.28)	(1.99–1.96)	(2.07–2.00)	(1.94–1.89)
Unique reflections	7330 (371)	34,696 (754)	69,859 (6889)	15,407 (747)
Completeness (%)	100.0 (100.0)	85.9 (38.2)	100.0 (100.0)	99.5 (90.1)
Mean I/sigma(I)	99.2 (2.1)	23.0 (2.3)	24.0 (3.5)	50.8 (3.0)
Redundancy	3.8 (3.69)	11.3 (5.6)	1.3 (1.24)	7.7 (6.8)
*R*_*merge*_ (%)^a^	7.3 (3.4)	8.4 (6.74)	8.7 (68.7)	21.7 (98)
*R*_*measure*_ (%)^b^	8.7 (1.34)	11.9 (10.3)	11.9 (70.9)	9.8 (91.8)
*R*_*pim*_ (%)^b^	1.4 (3.2)	3.4 (12.7)	3.3 (2.0)	1.2 (10.4)
CC_1/2_ (%)	99.7 (87.8)	100 (47.1)	99.4 (88.3)	100.1 (97.4)
Refinement statistics				
Resolution range (Å)	49.01–2.28	34.21–1.96	30.61–2.00	27.45–1.89
*R*_*work*_/*R*_*free*_ (%)^c^	23.4/26.6	17.9/23.7	19.8/20.1	20.5/23.6
Protein atoms	1153	3495	6708	1135
Ligand atoms	None	87	120	29
Water atoms	14	367	480	158
R.m.s. deviations				
Bond lengths (Å)	0.65	0.66	0.4	0.76
Bond angles (°)	0.89	0.83	0.59	0.88
Ramachandran plot (%)				
Favored	96	97	98	98
Allowed	100	98	100	100
Outliers	0	2	0	0
*PDB code*	6LJO	6LJ3	6LIS	6LJJ

Values in parentheses represent the highest resolution shell.

a *R*_*merge*_*= Σ*_*hkl*_*Σ*_*i*_*|I(hkl)*_*i*_*-<I(hkl)>|/Σ*_*hkl*_*Σ*_*i*_*I(hkl)*_*i*_, where *I(hkl)* is the intensity of reflection *hkl* and its symmetry equivalents and *<I(hkl)>* is the average intensity over all equivalent reflections.

b *R*_*measure*_: multiplicity-weighted *R*_*merge*_; *R*_*pim*_: precision-indicating *R*_*merge*_.

c *R = Σ||Fo| − |Fc||/Σ|Fo|*. *|Fo|* and *|Fc|* are amplitudes of the observed and calculated structure factors, respectively. *R*_*work*_ is the *R* value for reflections used in the refinement, whereas *R*_*free*_ is the *R* value for 5% of the reflections, which are selected in thin shells and are not included in the refinement.

The swine dUTPase (sDUT) gene consists of 663 bp and encodes 221 amino acid residues (Fig. 1*E*). The prokaryotic expression of full-length swine dUTPase was difficult, so an N-terminal truncated form of sDUT (residues 54–221) was expressed. Fortunately, we obtained the swine dUTPase trimeric structure in the complex structure of sDUT-dUPNPP-Mg^2+^ (PDB: 6LJJ). The asymmetric unit contained three subunits with the clear electron density of the C terminus. The secondary structure distribution of swine dUTPase is clearly presented (Fig. 1*C*), with residues Arg^30^-Arg^141^ forming the β-barrel core; β2-β5, α1, and β7-β12 forming a stable β-barrel; and β1 and β6 capping the barrel. β-strand β13 extends outward form the subunit and contributes a β-strand arm to the adjacent subunit, and the three active sites are located at the interface between adjacent subunits (Fig. 1*D*).

### Structural diversity of assembly active sites in the trimeric dUTPase family

3D domain swapping is a mechanism for forming oligomeric proteins from subunits. One example is dUTPase (24, 25). dUTPase structures uploaded to the PDB, which are usually homotrimeric oligomers, have been reported in detail (19). The classical trimer dUTPase possesses the following three types of major intersubunit interactions: (i) extended bimolecular interfaces, (ii) threefold central channels, and (iii) C-terminal β-strand swapping (9, 26).

**Figure 2:**
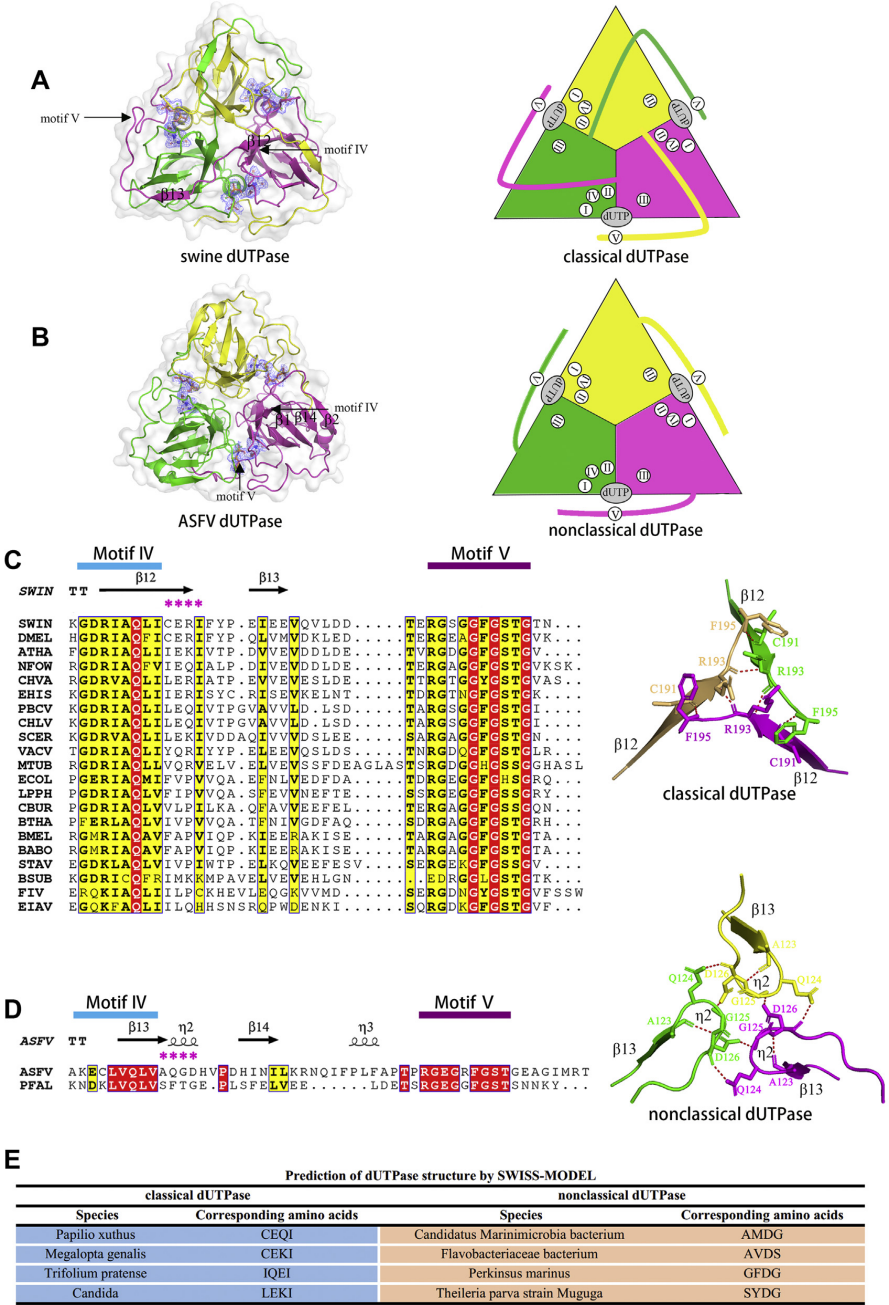
**Comparison of the assembly methods of swine dUTPase and ASFV dUTPase.***A*, top view of the swine dUTPase. The complex crystal structure (*left*) and schematic cartoon (*right*) are also shown. *B*, top view of the ASFV dUTPase. *C*, sequence alignment of Motifs IV and V in classical dUTPases. The four amino acids behind motif IV are marked with *purple stars*. *D*, sequence alignment of Motifs IV and V in nonclassical dUTPases. *E*, prediction of eight dUTPase structures.

**Figure 3:**
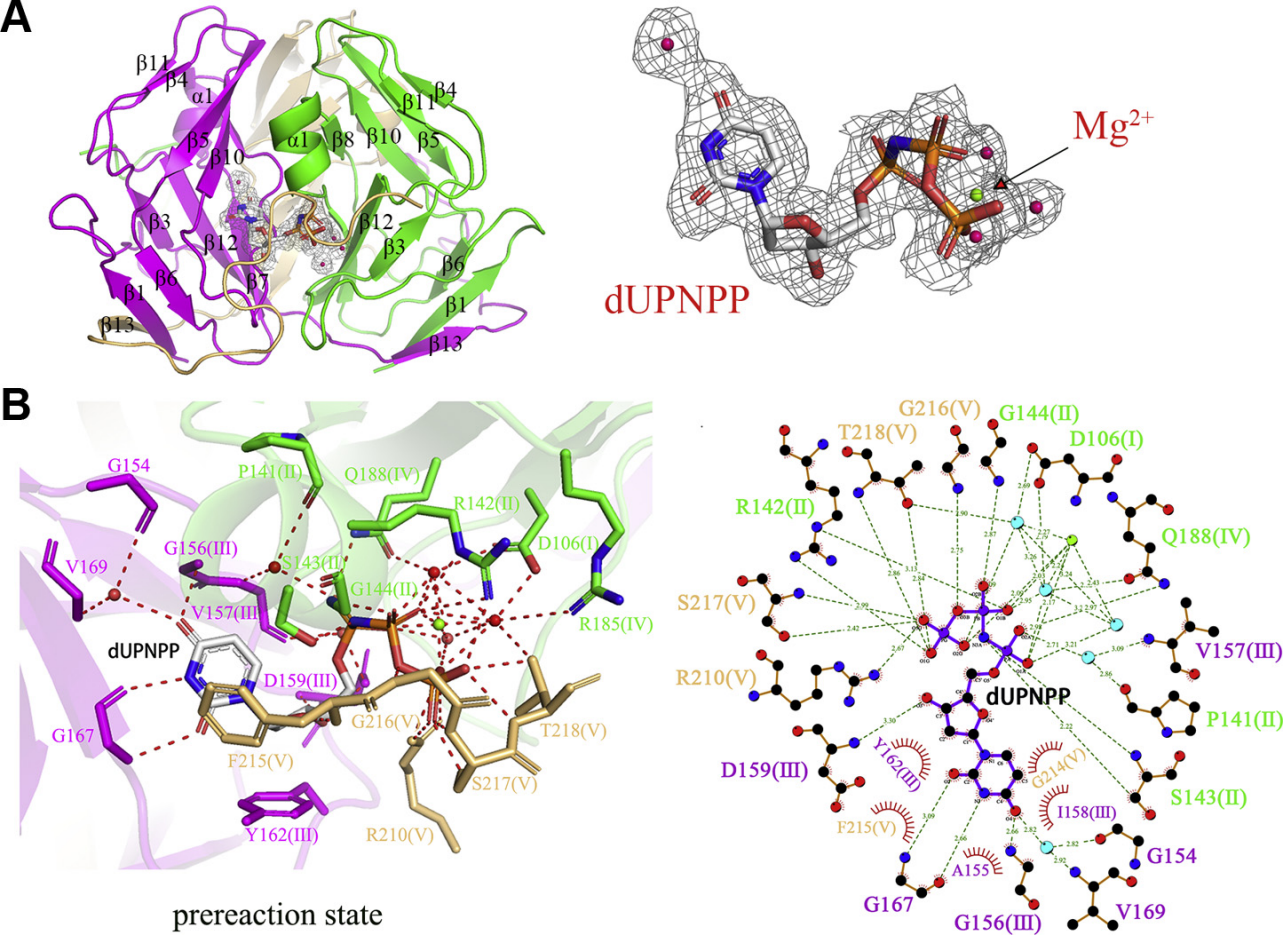
**The classical active sites in swine dUTPase.***A*, display substrates and electron densities in swine dUTPase. *B*, protein–ligand interactions in the sDUT-dUPNPP-Mg^2+^ structure active site. The active site of swine dUTPase is composed of residues from three subunits. Amino acid residues are colored by subunits.

The swine dUTPase active site is inseparable from the trimer assembly as follows: one subunit provides catalytic motifs I, II, and IV; another subunit provides motif III; and another subunit provides motif V (Figs. 2*A* and 3*A*). In detail, as β12 is antiparallel to β3 and β7, the C-terminal β-strand (β13) is swapped to the adjacent subunits, and eventually, the C-terminal motif V reaches the active site opposite of its own subunit (Fig. 2*A*). Therefore, swine dUTPase is a classical dUTPase (Fig. 2*A*).

**Figure 4:**
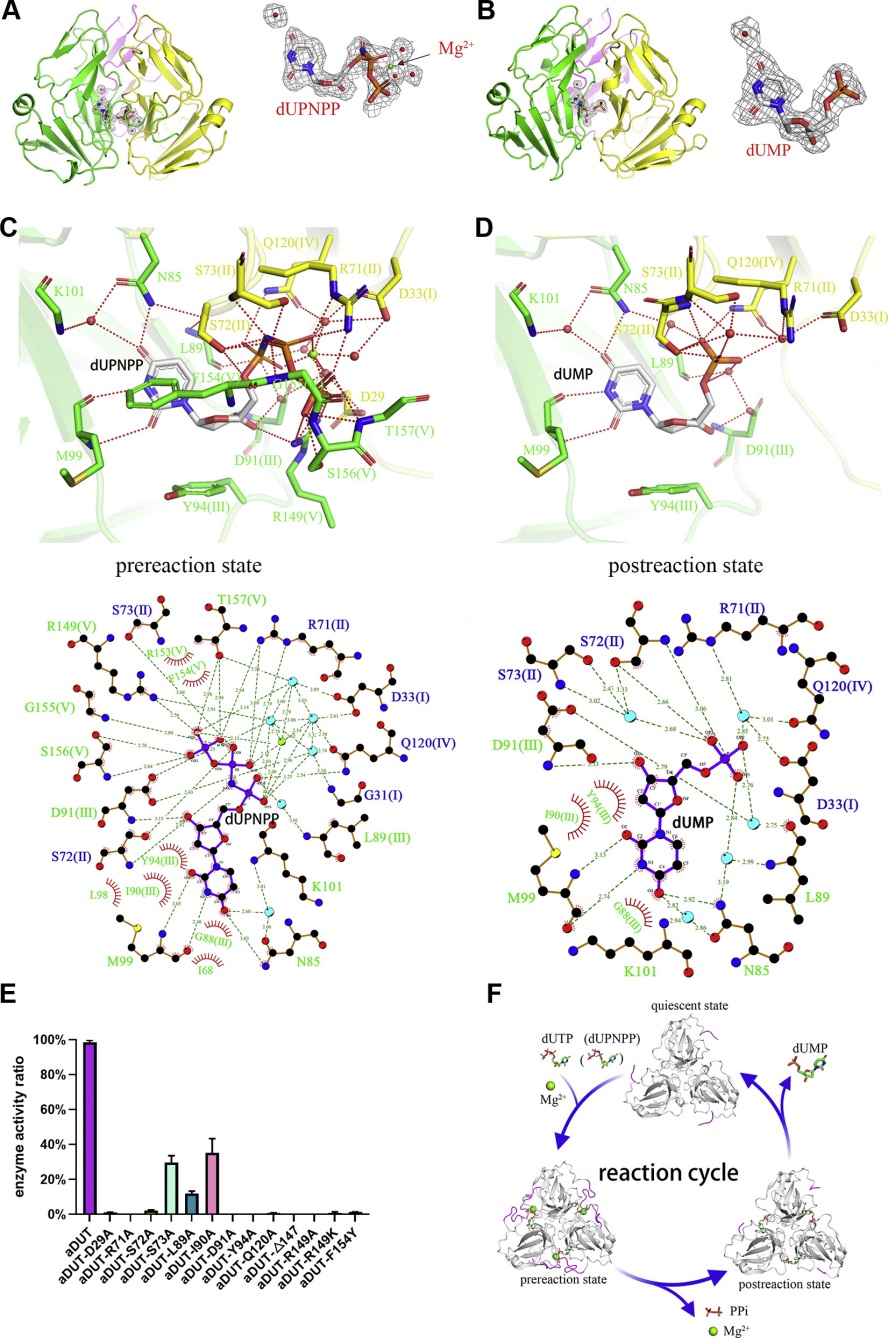
**ASFV dUTPase adopts a double-subunit active site for substrate catalysis.***A*, the dUPNPP, Mg^2+^, and electron densities in ASFV dUTPase. The σA-weighted 2Fo − Fc electron density maps (contoured at 0.5σ level) are shown for the substrates in the complex structures. The water molecules are indicated by *red balls*. *B*, the dUMP and electron densities in ASFV dUTPase. *C*, protein–ligand interactions in the aDUT-dUPNPP-Mg^2+^ structure active site. The active site of ASFV dUTPase is composed of residues from two subunits. The amino acid residues are colored by subunits with conserved motifs in parentheses. *D*, protein–ligand interactions in the aDUT-dUMP structure active site. *E*, effect of ASFV dUTPase (aDUT) active-site residue variants on catalysis. The relative efficiency was calculated as the enzyme efficiency (*K*_cat_/*K*_*m*_) of the mutants divided by that of the wildtype enzyme. *F*, the ASFV dUTPase reaction cycle. The flexibility of the C terminus is indicated in *magenta*.

However, surprisingly, there is no C-terminal β-strand swapping in ASFV dUTPase, and the aDUT-dUPNPP-Mg^2+^ structure provides definite evidence to support the presence of two-subunit active sites in the trimeric dUTPase. The ASFV dUTPase active site is formed by the following two subunits: one subunit provides catalytic motifs I, II, and IV and the other subunit provides motifs III and V (Figs. 2*B* and 4*A*). The C terminus (motif V) of ASFV dUTPase continues to reach the active site of its own subunit instead of reaching the third subunit as seen in a classical dUTPase (Fig. 2*B*). This novel orientation is attributed to C-terminal β-strand β14 not swapping, which suggests that ASFV dUTPase is a nonclassical dUTPase (Fig. 2*B*).

More interestingl, based on the comparison of classical dUTPases and nonclassical dUTPases in PDB, we found that whether the C-terminal β-strand is swapped is reflected in primary sequences. In classical dUTPases, the four amino acids behind motif IV (marked with purple star) are conserved; they are included as consisting of Glu, Gln, Cys, Pro, Arg, Ile, Val, and Leu in the library (Fig. 2*C*). For example, in swine dUTPase, the corresponding four amino acids are Cys, Glu, Arg, and Ile, and these motif IV–corresponding amino acids form a long β-strand (β12). The Arg^193^ at the end of β12 forms hydrogen bonds with an adjacent Arg^193^, and this rigid structure fixes the direction of subsequent amino acids, which ensures that the C-terminal β-strand (β13) is swapped into a neighboring subunit (Fig. 2*C*).

ASFV dUTPase and *Plasmodium falciparum* dUTPase are both nonclassical dUTPases (27). In nonclassical dUTPases, the four amino acids behind motif IV (marked with purple star) are also quite conserved; the conserved library consists of Ala, Asp, Gly, Ser, and Thr. Most of these residues are flexible amino acids, which provide a good degree of freedom for the development of the chain (Fig. 2*D*). For example, in ASFV dUTPase, the four corresponding amino acids are Ala, Gln, Gly, and Asp. In the secondary structure, the motif IV–corresponding amino acids form a short β-strand (β13). Then, η2, which consists of residues Ala^123^ to Asp^126^, leads to chain rotation *via* hydrogen bonds, and the C-terminal β-strand (β14) turns and inserts into the barrel of its own subunit, eventually bringing catalytic motif V into the active site. The C-terminal tail covers the rather flat surface of the trimer body and exhibits a meandering conformation (Fig. 2*D*).

Phylogenetic analysis was performed based on the multiple alignment using the MEGA package (28). The phylogenetic tree shows that the primary sequences of dUTPases from different species are divided into two clusters (Fig. S1), with each cluster corresponding to a dUTPase type (classical dUTPase and nonclassical dUTPase). The branching pattern of classical dUTPases coincides with other previously published phylogenetic analyses (29). However, the presence of the ASFV dUTPase and *P. falciparum* dUTPase sequences change the global pattern, with the nonclassical dUTPase sequences grouping together in a separate branch (Fig. S1).

Therefore, the following are two factors to predict whether a dUTPase is a classical or nonclassical dUTPase: (i) the four amino acids behind motif IV and (ii) the phylogenetic analysis. We selected dUTPases from eight species to verify our conclusion. Their structures have not been resolved, although the four amino acids behind motif IV belong to the respective libraries (Fig. 2*E*). Phylogenetic analysis showed that the primary sequences of the eight species’ dUTPases are also divided into two clusters (Fig. S2). According to our classification results, the eight dUTPase structures from the different species are consistent with the results predicted by SWISS-MODEL.

### Swine dUTPase adopts the classic three-subunit active sites to bind substrates

In contrast to ASFV dUTPase, swine dUTPase (sDUT) is a classical dUTPase. The sDUT-dUPNPP-Mg^2+^ complex structure represents the prereaction state of the substrates just after binding to the active site (Fig. 3*A*). In the sDUT-dUPNPP-Mg^2+^ complex structure (Fig. 3*B*), (i) π-π stacking interactions are observed between the “Phe-lid” Phe^215^ and the uracil ring; (ii) hydrogen bonding is observed between Gly^167^, Gly^156^, Val^169^, and Gly^154^ and the uracil ring; (iii) stacking interactions are observed between Tyr^162^ (a highly conserved tyrosine found within dUTPases) and the deoxyribose; (iv) Asp^159^ appears well poised to abstract a proton from an active site water, activating it for nucleophilic attack on the alpha-phosphate, and is also positioned to discriminate against deoxyribose by the presence of the 3’-OH group; (v) Mg^2+^, the oxygen atoms on three phosphate groups, and three water molecules form a hexa-coordinate complex; (vi) the β- and γ-phosphates interact with Gly^144^, Gly^216^, Ser^217^, and Thr^218^; and (vii) Arg^210^ is the arginine finger (Fig. 3*B*). There is 90% genetic similarity between swine dUTPase and human dUTPase, and their five conserved motifs were also consistent. Michaelis–Menten-type substrate kinetics analysis indicated that swine dUTPase has a *K*_*M*_ of 0.58 ± 0.13 μM and a *K*_cat_ of 5.58 ± 0.6 s^−1^ (Table 2).

**Table 2:** Enzyme kinetics raw data for ASFV dUTPase and swine dUTPase

Enzyme	*Km* (μM)	*K*_cat_ (s^−1^)	*k*_cat_*/Km* (M^−1^ s^−1^)	Relative efficiency (%)
aDUT^WT^	0.98 ± 0.2	4.37 ± 0.15	4.46 × 10^6^	100%
aDUT^D29A^	6.89 ± 0.2	0.31 ± 0.1	4.62 × 10^4^	1.04%
aDUT^R71A^	∖	∖	∖	∖
aDUT^S72A^	4.89 ± 0.35	0.48 ± 0.2	9.85 × 10^4^	2.20%
aDUT^S73A^	4.16 ± 0.19	5.58 ± 0.3	1.34 × 10^6^	29.99%
aDUT^L89A^	5.44 ± 0.5	2.94 ± 0.7	0.54 × 10^6^	34.17%
aDUT^I90A^	0.47 ± 0.26	0.75 ± 0.2	1.58 × 10^6^	35.37%
aDUT^D91A^	∖	∖	∖	∖
aDUT^Y94A^	∖	∖	∖	∖
aDUT^Q120A^	16.76 ± 1.21	0.58 ±0.4	3.51 × 10^4^	0.79%
aDUT^△147^	∖	∖	∖	∖
aDUT^R149A^	∖	∖	∖	∖
aDUT^R149K^	4.51 ± 0.7	0.15 ± 0.1	0.33 × 10^5^	0.73%
aDUT^F154Y^	26.45 ± 0.2	1.5 ± 0.1	0.56 × 10^5^	1.26%
sDUT^WT^	0.58 ± 0.13	5.58 ± 0.6	9.60 × 10^6^	100%

‘∖’: no activity or diminished the enzymatic activity to an undetectable level.

### ASFV dUTPase employs nonclassical dual-subunit active sites

ASFV dUTPase (aDUT) is a nonclassical dUTPase. The aDUT-dUPNPP-Mg^2+^ complex structure represents the prereaction state of the substrates just after binding to the active site (Fig. 4*A*). The protein-ligand interactions for ASFV dUTPase (aDUT) were elucidated in great detail (Fig. 4*C*). To facilitate understanding, a two-dimensional representation of the protein-ligand interactions is presented below. In the aDUT-dUPNPP-Mg^2+^ complex structure (prereaction state), (i) π-π stacking interactions are formed between the Phe-lid Phe^154^ and the uracil ring; (ii) hydrogen bonding is observed among Met^99^, Lys^101^, Asn^85^, and the uracil ring; (iii) stacking interactions are formed between Tyr^94^ and the deoxyribose; (iv) Asp^91^, a recognized key site, is involved in an α-phosphate nucleophilic attack; (v) α-phosphates interact with Ser^72^ and Gln^120^; β-phosphates interact with Arg^71^, Ser^72^, Ser^73^, and Gly^155^; and γ-phosphates interact with Arg^71^, Ser^73^, Gly^155^, Ser^156^, and Thr^157^; (vi) the position of Arg^149^, as the arginine finger, interacts with a γ-phosphate; (vii) Mg^2+^, oxygen atoms on three phosphate groups, and three water molecules form a hexa-coordinate complex; and (viii) the C terminus (motif V), like a lid, was in a closed state to provide the microenvironment for the active site.

The aDUT-dUMP structure represents the postreaction state of the product dUMP bound to the active site (Fig. 4*B*). The crystal of the aDUT-dUMP complex grew in the P2_1_2_1_2_1_ space group with two trimers consisting of six subunits in each asymmetric unit (Table 1). All of the subunits in the asymmetric unit have essentially identical structures, although the C terminus lacks clear electron density. In the aDUT-dUMP complex structure (postreaction state) (Fig. 4*D*), (i) dUMP tightly bound to key amino acids in aDUT in the same manner as dUPNPP, Met^99^ was responsible for pairing with the uracil ring, and Tyr^94^ was responsible for the recognition and stabilization of deoxyribose; (ii) α-phosphates interact with Arg^71^ and Ser^72^; and (iii) the C terminus (motif V) returned to an open state, which is very conducive to product release (Fig. 4*D*).

Comparison of the prereaction state and quiescent state showed a root-mean-square deviation (RMSD) of 0.519 Å for the active sites. These differences are evidence of the conformational changes induced by dUTP binding, as active-site residues are rearranged to provide a tight pocket for the substrate (Table S1). Comparison of the postreaction state and quiescent state showed an RMSD of 0.364 Å for the active sites, suggesting that the conformational changes are also beneficial to the release of the product (Table S1).

**Figure 5:**
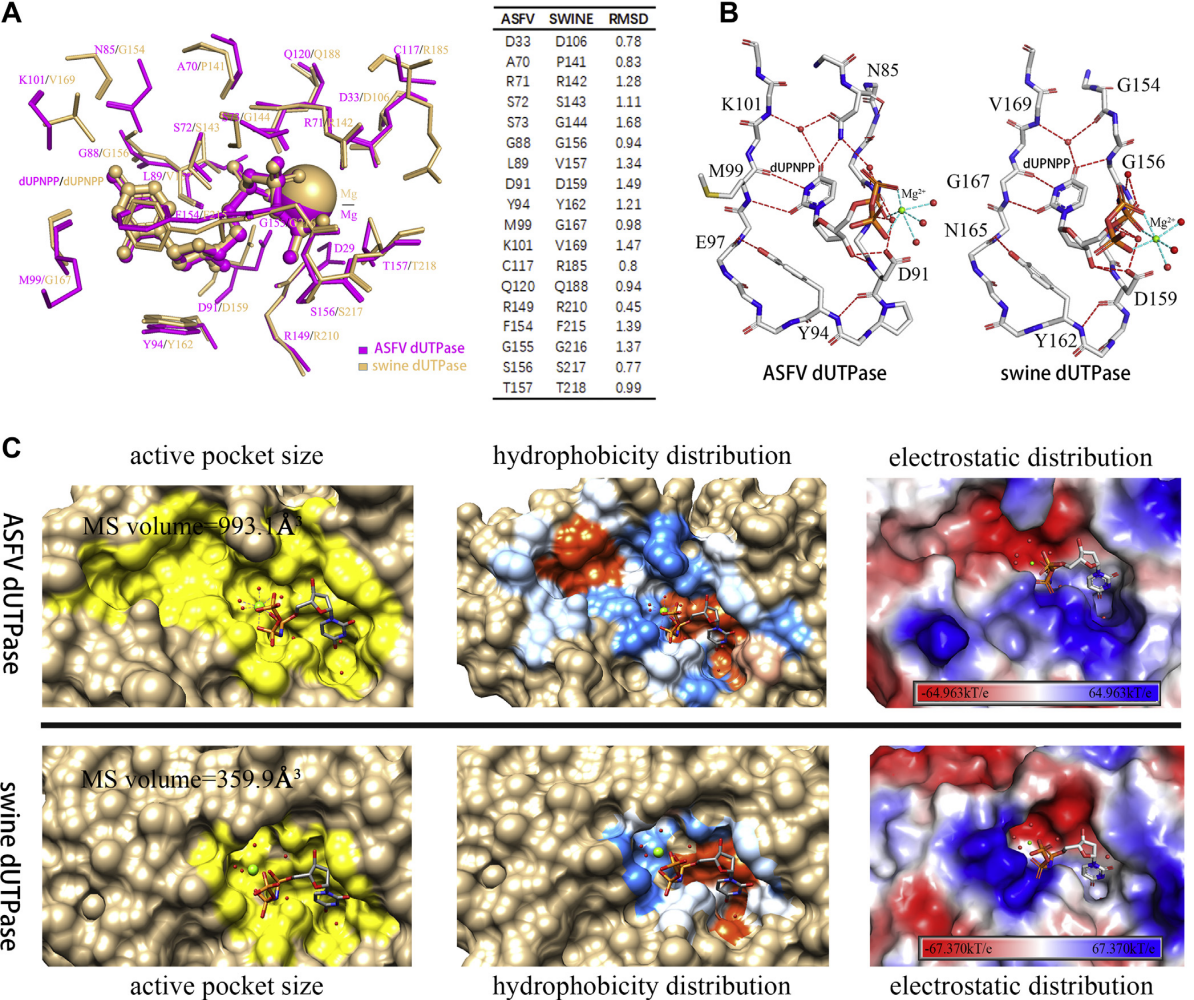
**Comparison of the active pockets of ASFV dUTPase (aDUT) and swine dUTPase (sDUT).***A*, superimposition of the active sites of aDUT (magenta) and sDUT (light orange). Amino acid residues, water molecules, amino acid residues, and Mg^2+^ are colored by source. RMSD values of the residues in ASFV dUTPase and swine dUTPase. *B*, both ASFV dUTPase and swine dUTPase use tight β hairpin-like structures to identify and bind substrates. Hydrogen bonds are indicated by *red dotted lines*. The six coordination bonds formed with Mg^2+^ are indicated by *cyan dotted lines*. *C*, comparison of the volume, hydrophobicity, and electrostatics of the active site pockets of aDUT and sDUT. Hydrophilicity, *dodger blue*. Hydrophobicity, *orange red*. Positive electrostatic region, blue. Negative electrostatic region, *red*. The active sites assign protonation states at pH 7.0 using the PDB2PQR and APBS software (k, the Boltzmann constant; *T*, temperature; e, the charge of an electron).

With respect to the amino acids, the ASFV dUTPase active site shares structural similarity with that of a classical dUTPase (Fig. 5*A*), indicating that ASFV dUTPase maintains the same catalytic mechanism as the classical dUTPase, even though its active site assembly method is different from a classical dUTPase. A schematic cartoon is depicted in Figure 4*F*. When dUTP and Mg^2+^ enter the active site pocket, the uracil ring forms hydrogen bonds with the main-chain oxygen and nitrogen of Met^99^, and the resultant hydrogen bonds can recognize and fix the uracil ring by imitating Watson–Crick pairing. Tyr^94^, located at the bottom of the active site, can discriminate the ribose by stacking with deoxyribose. The C terminus (motif V) changes to a closed state, closing the active site (Fig. 4*C*). Phe^154^ is conjugated with uracil to further fix the uracil ring. One water molecule, coordinated by the strictly conserved Asp^91^, is fixed for nucleophilic attack on the α-phosphate group. dUTP is hydrolyzed and Arg^71^ might play a key role in neutralizing the negative charge in the catalytic process. The Arg^149^ side-chain guanidino group forms a hydrogen bond with a γ-phosphate, and nucleophilic attack occurs on the α-phosphate in dUTPases; therefore, the arginine finger corresponding to Arg^149^ is not directly involved in electronic stabilization (Fig. 4*C*). Mg^2+^ is responsible for neutralizing the repulsion between the negative charges generated by the PPi and dUMP leaving groups during hydrolysis. After the catalytic reaction, the C terminus (motif V) returns to an open state (Fig. 4*F*), and PPi at the entrance of the active site is expected to exit freely since it can easily interact with the solvent. The magnesium ion (charge 2+) might facilitate PPi discharge since it is more likely to bind PPi (charge −4) than dUMP (charge −2). The arginine finger (Arg^149^) might also participate in PPi escape *via* charge stabilization.

Michaelis–Menten-type substrate kinetics analysis indicated that ASFV dUTPase has a *K*_*M*_ of 0.98 ± 0.2 μM and a *K*_cat_ of 4.37 ± 1.5 s^−1^ (Table 2). A mutagenesis study of ASFV dUTPase was carried out to test the roles of individual active-site residues in catalysis. (i) Deletion of motif V (aDUT^Δ147^) abolished the catalytic ability, demonstrating its crucial role that has been well characterized in other dUTPases (9, 30). (ii) Mutation of residues Asp^91^, Arg^71^, Tyr^94^, and Arg^149^ into alanine resulted in the complete lack of enzyme activity or diminished enzymatic activity to an undetectable level (Fig. 4*E* and Table 2), preventing measurement. These findings suggest the important role of amino acids in the catalytic process. Mutating the catalytic residue Asp^91^, thereby removing its role as a general base in catalysis, also eliminated enzyme activity. Mutating Arg^71^ affected the neutralization of the negative charge generated by the catalytic process. Mutating Tyr^94^ reduced the affinity with the substrates. Replacing Arg^149^ may damage the arginine finger that organizes the active-site conformation.

(iii) Mutation of residues Asp^29^, Arg^72^, Ser^73^, and Leu^89^ into alanine resulted in an approximately 4- to 7-fold increase in the *K*_*m*_ in these mutants (aDUT^D29A^ *K*_*M*_ = 6.89 ± 0.2 μM, aDUT^R72A^ *K*_*M*_ = 4.89 ± 0.35 μM, aDUT^S73A^ *K*_*M*_ = 4.16 ± 0.19 μM, and aDUT^L89A^ *K*_*M*_ = 5.44 ± 0.5 μM), supporting a critical role for Asp^29^ and Arg^89^ in substrate recognition (Fig. 4*E* and Table 2). Arg^72^ and Ser^73^ participate in binding to phosphate groups, and mutations would result in reduced affinity of the active center to the substrate. (iv) Mutation of Gln^120^ into alanine resulted in a more than 100-fold reduction in the catalytic efficiency owing to an increase in the *K*_*M*_ (*K*_*M*_ = 16.76 ± 1.21 μM, *K*_cat_ = 0.58 ± 0.4 μM); therefore, dUTP binding affinity was notably decreased. (v) Mutation of Ile^90^ into alanine caused a significant decrease in the *K*_cat_ (*K*_*M*_ = 0.47 ± 0.26 μM and *K*_cat_ = 0.75 ± 0.2 μM), suggesting the critical role of Ile^90^ in maintaining appropriate interactions at the active site. (vi) Mutation of Phe^154^ to tyrosine caused an observable reduction in both the catalytic rate and substrate-binding affinity (*K*_*M*_ = 26.45 ± 0.2 μM and *K*_cat_ = 1.5 ± 0.1 μM, respectively) (Fig. 4*E* and Table 2).

### Similarities and differences between the active sites of ASFV dUTPase and swine dUTPase

Targeted inhibitor development will focus on analyzing the similarities and differences between ASFV dUTPase and swine dUTPase. Superposing of ASFV dUTPase and swine dUTPase gives an RMSD of 0.801 Å for the active sites and 1.917 Å for the overall structures (Table S1). We also compare the amino acid responsible for binding to substrate and calculate its RMSD value; key amino acids in the ASFV dUTPase and swine dUTPase active sites are almost identical, but the RMSD value of conservative amino acids is around 1.0 Å, with five amino acids Arg^71^, Asn^91^, Tyr^94^, Phe^154^, and Gly^155^ whose RMSD value exceed 1.2 Å. Some nonconservative amino acids, such as Pro^141^ and Arg^185^ in swine dUTPase and Lys^101^ in ASFV dUTPase, also form hydrogen bond interactions with dUPNPP (Fig. 5*A*); for example, in ASFV dUTPase, the amino acids that form hydrogen bonds with the uracil ring *via* water molecules are Lys^101^ and Asn^85^, whereas in swine dUTPase, these amino acids are Val^159^ and Gly^154^. Moreover, both Met^99^ of ASFV dUTPase and Gly^167^ of swine dUTPase offer main-chain oxygen and nitrogen atoms rather than side-chain atoms for hydrogen bonds with the uracil (Fig. 5*A*).

The mechanisms by which NTPs and dNTPs are prevented from binding the active site are identical in ASFV dUTPase and swine dUTPase (Fig. 5*B*). On the one hand, the residues in motif III lead to steric hindrance, preventing purines, thymine, and the ribose from binding to the active sites. A tight β hairpin-like structure bounded uracil and deoxyribose excludes the purines and thymine. A tyrosine (Tyr^94^ of ASFV dUTPase and Tyr^162^ of swine dUTPase) at the bottom of the β hairpin–like structure is responsible for stacking with the deoxyribose and excluding ribose. In contrast, hydrogen bonds at the uracil were found by imitating base pairing, as the uracil ring forms hydrogen bonds with the Met^99^ of ASFV dUTPase or Gly^167^ of swine dUTPase, thereby mimicking Watson–Crick pairing (Fig. 5*B*).

As calculated by CASTp (31), the molecular surface volume in ASFV dUTPase is 993.1 Å^3^. However, in swine dUTPase, the molecular surface volume is 359.9 Å^3^, and the pocket in the former is almost three times as large as that in the latter (Fig. 5*C*). In addition, there was no significant difference in the hydrophobicity distribution of ASFV dUTPase and swine dUTPase; the hydrophobic region contributes to fixation of the uracil ring and deoxyribose, and the hydrophilic region facilitates stabilization of the phosphates in the active sites (Fig. 5*C*). However, their electrostatic distributions differed, especially the position of the uracil ring in the active site pockets. In ASFV dUTPase, this position is in a completely positive charge region, but this position is uncharged in swine dUTPase (Fig. 5*C*).

### ASFV dUTPase tolerates higher temperatures

**Figure 6:**
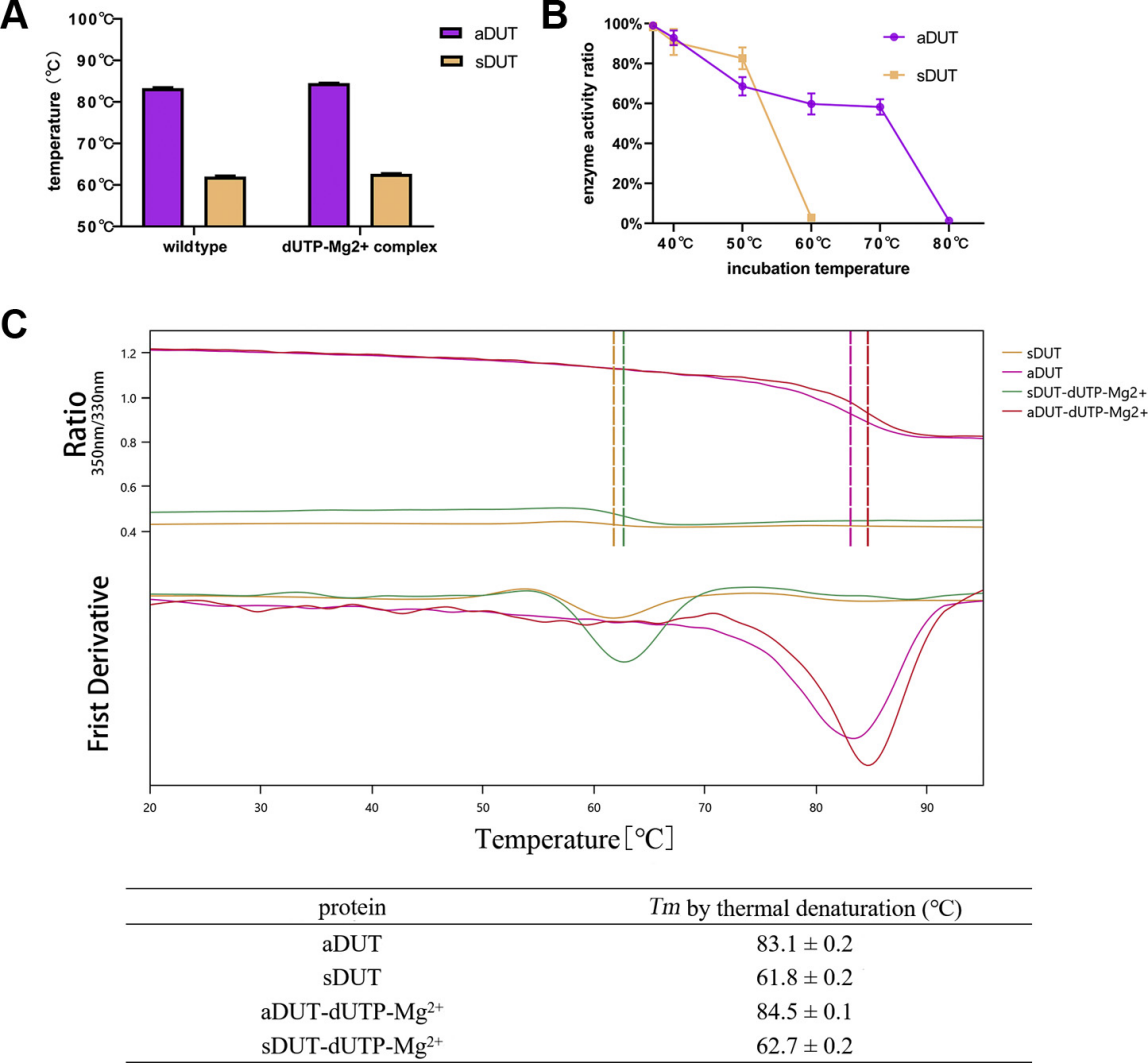
**Results of thermofluor and thermal inactivation assays of ASFV dUTPase and swine dUTPase.***A*, the thermal stability of ASFV dUTPase (aDUT) and swine dUTPase (sDUT). *B*, the thermal inactivation of ASFV dUTPase and swine dUTPase. *C*, raw data from the thermofluor assay.

The results of the thermofluor assay showed that ASFV dUTPase was denatured when heated to approximately 83 °C, whereas swine dUTPase denatured at 62 °C, indicating that ASFV dUTPase had better thermal stability (Fig. 6*A*). When dUTP and Mg^2+^ were added to the reaction system in separate heating cycles, the *T*_*m*_ value was increased by approximately 2 °C owing to the addition of these substrates (Fig. 6*C*). The thermal inactivation of ASFV dUTPase and swine dUTPase was measured after 10 min of incubation at different temperatures, and these results showed that the enzyme activity of swine dUTPase decreased rapidly with increasing temperature and the enzyme lost its activity at 60 °C. The ASFV dUTPase remains active at higher temperatures, and as the temperature increases, it can still maintain stable activity within a temperature gradient. Its deactivation temperature reaches 80 °C (Fig. 6*B*), similar to the application of high-temperature–resistant DNA polymerase in PCR assays. This specific high temperature resistance of ASFV dUTPase means that it has great prospects for development into an enzymatic tool, for example, for removing dUTP in nucleotide libraries *in vitro*.

### Inhibitory activity of compounds and molecular docking model

**Figure 7:**
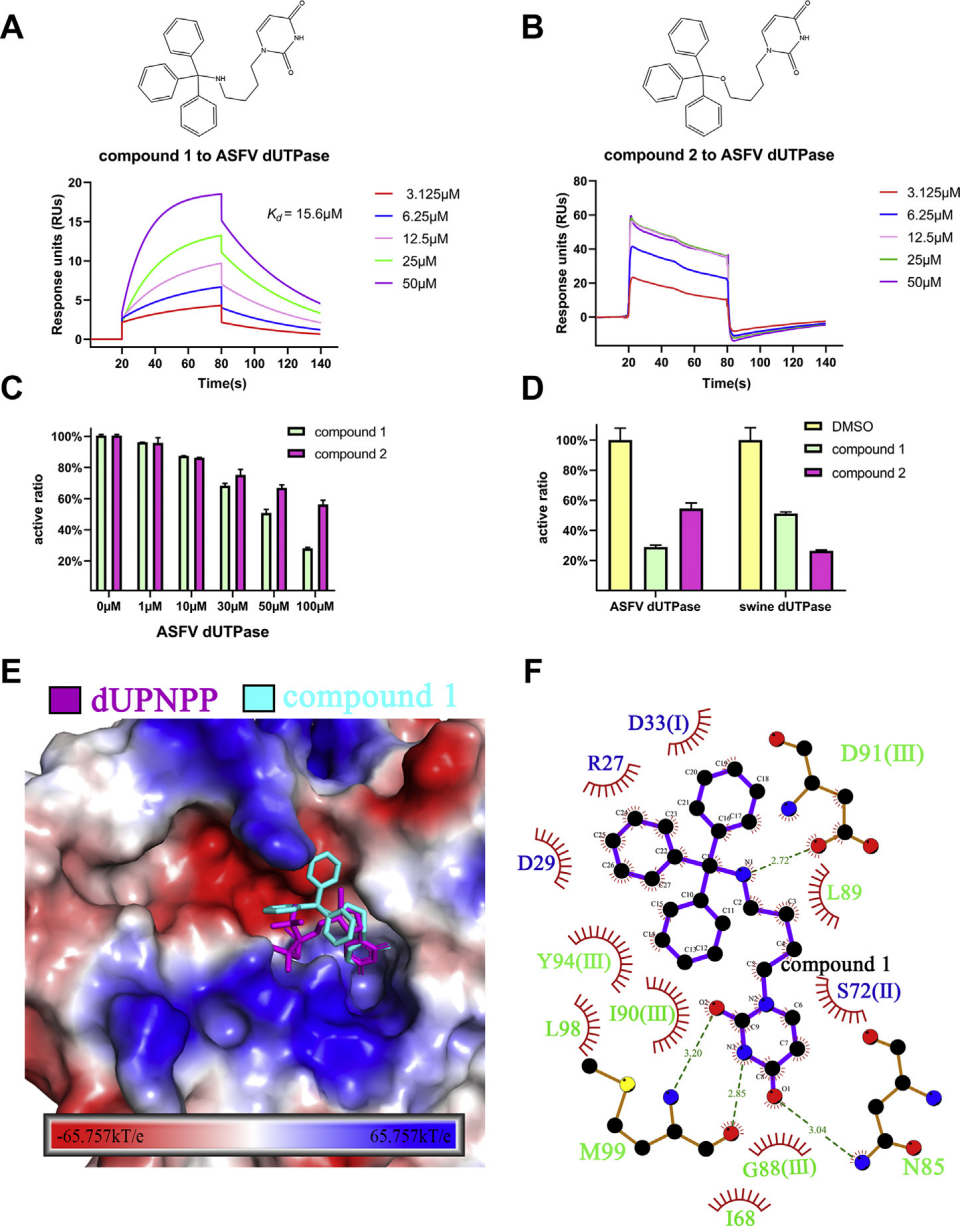
**Initial molecular modeling with the inhibitor of ASFV dUTPase.***A*, structure of the inhibitor compound 1 and surface plasmon resonance assay characterizing the ASFV dUTPase/compound 1 binding kinetics. Gradient concentrations of compound 1 were flow through ASFV dUTPase immobilized on the chip surface. The real-time binding profiles are recorded and shown. *B*, structure of the inhibitor compound 2 and surface plasmon resonance assay characterizing the ASFV dUTPase/compound 2 binding kinetics. *C*, enzyme activity of ASFV dUTPase was inhibited by compounds 1 and 2 in a dose-dependent manner. *D*, analysis of inhibition of ASFV dUTPase and swine dUTPase enzyme activity. *E*, docking pattern of the interaction between inhibitor compound 1 and ASFV dUTPase. The electrostatic distribution mapped to the solvent-accessible surface of ASFV dUTPase. Positive electrostatic region, *blue*. Negative electrostatic region, *red*. *F*, protein–inhibitor interactions in the ASFV dUTPase active site.

The structure of ASFV dUTPase is similar to that of *P. falciparum* dUTPase; swine dUTPase is similar to human dUTPase; and the targeted inhibitor of pathogen (*P. falciparum*) dUTPase and host (human) dUTPase can be used as a good model for reference. We synthesized two compounds (1 and 2) that were designed for *P. falciparum* dUTPase, the structures of compounds were confirmed by NMR spectra, and the purity of compounds was determined by HPLC analysis to be ≥95% (Figs. S3 and S4). SPR analyses suggested that compound 1 acts as a competitive inhibitor with a *K*_*d*_ of 15.6 μM against ASFV dUTPase (Fig. 7*A*), whereas compound 2 quickly dissociates from ASFV dUTPase during the binding phase, so its *K*_*d*_ cannot be calculated (Fig. 7*B*). The inhibition kinetics measurement results showed that enzyme activity of ASFV dUTPase was inhibited by compounds 1 and 2 in a dose-dependent manner. Compound 1 is better for inhibiting ASFV dUTPase than compound 2; this is consistent with SPR results (Fig. 7*C*). Interestingly, inhibitory activity of compound 1 was selective for ASFV dUTPase over swine dUTPase (Fig. 7*D*).

To further explore how compound 1 inhibits the enzyme activity of ASFV dUTPase, we docked the inhibitor to the active sites by using autodock (32). In general, it may be observed that compound 1 binds in a manner similar to which dUPNPP was found to bind crystallographically (Fig. 7, *E*–*F*). The uracil moiety of compound 1 positioned in the bottom of the active site, forming hydrogen bonds with Met^99^ and Asn^85^; the tributylamine group could form hydrogen bonds with Asp^91^. The residues of active sites like Asp^29^, Ile^68^, Leu^89^, Ile^90^, and Tyr^94^ might form hydrophobic interaction force with the candidate inhibitor, which will further stabilize the complex structure. As a result, compound 1 blocked dUTP into the enzyme active center and played an inhibitory activity, so compound 1 was an effective candidate inhibitor.

## Discussion

To date, there have been no effective treatments for ASFV. Thus, a suitable antiviral drug and an effective vaccine are urgent (1, 2). The results of a previous study showed that dUTPase deletion from ASFV could directly affect viral replication in macrophages; thus, ASFV dUTPase can serve as a potential antiviral target (14, 16, 33).

In this study, we determined the crystal structures of ASFV dUTPase in the pre- and postreaction states. Based on our results, we concluded that ASFV dUTPase is a nonclassical dUTPase, and we elaborated on nonclassical dual-subunit active sites. Moreover, we first reported the crystal structures of swine dUTPase and concluded that swine dUTPase is a classical dUTPase. We further explored the assembly between classical dUTPases and nonclassical dUTPases. Interestingly, we found that the type of a trimeric dUTPase can be predicted by sequence comparison and evolutionary tree analysis, which will provide broader implications for the dUTPase family.

We performed two candidate inhibitors against ASFV dUTPase and identified compound 1 that was active against ASFV dUTPase over swine dUTPase. These results suggested that inhibitor recognition specificity of ASFV dUTPase may differ from that of swine dUTPase, even though the overall structures of the whole protein and the catalytic sites are similar. To prevent the possible high cytosolic toxicity of inhibitors or drugs toward host swine, we revealed crucial structural differences between these two dUTPase enzymes and provided insights for developing inhibitors against ASFV: (i) The volume of substrate binding pockets of ASFV dUTPase is almost three times larger than that of swine dUTPase; adding rigid groups to the butyl of inhibitors to form steric resistance is a good strategy to prevent inhibitors from acting on swine dUTPase. (ii) An inhibitor could be designed based on the different electrostatic distributions and hydrophobicity distributions of the active sites. (iii) Adding groups on the butyl of compound 1 improved the affinity with the active sites. According to previous studies on dUTPase inhibitors, a flexible inhibitor (containing a flexible linker moiety) could be a promising agent to increase the affinity between inhibitors and target dUTPases (34, 35), because a flexible inhibitor could more easily adopt a conformation to minimize entropic loss (34). These observations should be considered in the development of inhibitors against ASFV dUTPase without affecting swine dUTPase.

In summary, our study identifies a novel method of ASFV dUTPase assembly and clarifies the nonclassical dual-subunit active sites. We obtained the structure of swine dUTPase and revealed its interactions with substrates. The structural work from our study increases the awareness of classical dUTPases and nonclassical dUTPases. Furthermore, to prevent possible high cytosolic toxicity of inhibitors or drugs toward host swine, the inhibitory activity of compounds provides guidance for the development of specific inhibitors and targeted drugs against ASFV.

## Experimental procedures

### Plasmid construction

The ASFV dUTPase gene (E165R) was amplified by PCR with ASFV genomic DNA as the template using oligonucleotide primers (Table 3). Total RNA was extracted from swine testis cells, and after reverse transcription of the RNA, cDNA was used as a template to obtain the swine dUTPase gene by PCR (Table 3). Primers were designed by referring to the complete genome of *Sus scrofa* (sequence ID: XM_003353370.3). The amplified DNA fragment was purified and cloned into the PET42b vector by homologous recombination. To avoid the effects of protein labeling, each protein was constructed in two forms, namely, with an N-terminal histidine tag and a C-terminal histidine tag.

**Table 3:** The primers for PCR

Target primer		Sequence (5′–3′)	Bases (bp)
aDUT	forward	ATGGCAACAAATTTTTTTATTCAAC	25
aDUT	reverse	TTAAGTTCTCATAATCCCGGCCTCG	25
sDUT	forward	ATGAGTCTCAGCCGAGGTTGCCG	23
sDUT	reverse	TTAATTCGTTCCAGTGGAACC	21

### Protein expression and purification

The ASFV and swine dUTPases were transferred into the *Escherichia coli* BL21 (DE3) strain. Briefly, the cultures were grown in LB medium supplemented with 33 μg/ml kanamycin and incubated at 37 °C in a rotatory shaker until reaching an OD 600 = 0.6 to 0.7, after which IPTG was added to the LB to a final concentration of 1 mM. Then, growth was continued at 27 °C for approximately 7 h. After induction, cells were harvested by centrifugation at 4 °C for 10 min at 6000*g*, resuspended in phosphate-buffered saline (pH 7.4), and lysed under pressure.

To overexpress Se-Met-substituted ASFV dUTPase, recombinant strains from 20 ml of an overnight culture were inoculated in 1 L of fresh M9 medium supplemented with 50  μg/ml kanamycin until reaching an OD 600 = 0.7 to 0.8, after which 30 mg/l Se-Met was added to the medium. After growth of the strain at 37  °C for 1  h, the temperature was lowered to 18  °C, and protein expression was induced by the addition of IPTG to the medium at a final concentration of 1  mM. The induced cultures were then grown at 18  °C for an additional 18  h, and the cells were harvested by centrifugation and lysed under pressure.

The lysate was purified by centrifugation at 8500*g* for 45 min, and the cleared supernatant was loaded onto a HisTrap HP column (GE Healthcare) pre-equilibrated with buffer A (20 mM Hepes and 500 mM NaCl, pH 7.4). After being washed with 30 ml of buffer A, the His-tagged dUTPase was eluted from the cobalt affinity column using buffer B1 containing 20 mM Hepes (pH 7.5), 500 mM NaCl, and 500 mM imidazole. The eluted dUTPase was collected, concentrated, and finally purified with size-exclusion chromatography using a HiLoad 16/60 Superdex 200 column (GE Healthcare) equilibrated with buffer B2 (10 mM Hepes [pH 7.5] and 200 mM NaCl). The purified dUTPases were analyzed by SDS-PAGE. After analysis, the purest dUTPases were selected and concentrated to approximately 10 mg/ml as determined by the absorbance at 280 nm. Finally, they were stored at −80 °C for subsequent analysis.

### Crystallization and data collection

For crystallization, the purified protein was concentrated to approximately 12 mg/ml. The Se-Met-labeled ASFV dUTPase (aDUT) was crystallized *via* the sitting-drop vapor diffusion method at 20 °C. The initial conditions were optimized for crystallization. The best Se-Met-labeled aDUT crystals were obtained by subjecting drops consisting of 1 μl of reservoir solution (0.5 M NaH_2_PO4/K_2_HPO_4_, pH 6.9) and 1 μl of protein solution (6 mg/ml in 10 mM Hepes and 200 mM NaCl, pH 7.4) through vapor diffusion. Crystals of the aDUT-dUPNPP-Mg^2+^ complex were grown in drops containing 1 μl of protein solution (5 mg/ml protein + 10 mm dUPNPP + 8 mm MgCl_2_ incubated for 1 h at 4 °C) and 1 μl of reservoir solution (0.2 M sodium citrate and 24% (w/v) PEG 3350). aDUT-dUMP crystals were grown in drops containing 1 μl of protein solution (10 mg/ml protein + 10 mm dUTP + 8 mm MgCl_2_ incubated for 1 h at 4 °C) and 1 μl of reservoir solution (0.2 M sodium citrate and 24% [w/v] PEG 3350). Crystals of ligand-free swine dUTPase (sDUT) were grown in drops containing 1 μl of protein solution (9 mg/ml in 10 mM Hepes and 200 mM NaCl, pH 7.4) and 1 μl of reservoir solution (0.05 M cadmium sulfate hydrate, 0.1 M Hepes pH 7.5, and 1.0 M sodium acetate trihydrate). Crystals of the sDUT-dUPNPP-Mg^2+^ complex were grown in drops containing 1 μl of protein solution (8 mg/ml protein + 10 mm dUPNPP + 8 mm MgCl_2_ incubated for 1 h at 4 °C) and 1 μl of reservoir solution (0.2 M magnesium formate [pH 5.9] and 20% [w/v] PEG 3350).

Diffraction-quality crystals were flash-frozen in liquid nitrogen for storage. X-ray diffraction data were collected at the BL17U and BL19U beamlines at the Shanghai Synchrotron Radiation Facility at cryogenic temperature.

### Structure determination

All of the diffraction images were integrated, merged, and scaled using the HKL-2000 software (36). The structure of aDUT was resolved from a Se-Met derivative using the single-wavelength anomalous-dispersion method. All three potential selenium atoms from the primary sequence of the ASFV dUTPase were located, and initial phases were calculated using the AutoSol program from the PHENIX software suite (37). The structure of the ligand-free sDUT was resolved by molecular replacement using the Phaser program (38) with *Homo sapiens* dUTPase (PDB: 2HQU) as a model. The ligand-bound structures were resolved by molecular replacement with the refined ligand-free structure used as a model. Ligands were modeled at a late stage of refinement, and the geometry restraints of the ligands were generated with the GRADE server (http://grade.globalphasing.org). Manual model rebuilding was performed using COOT (39), and subsequently, the structures were refined by using the PHENIX software suite. Sequence alignment was performed on the ESPript server (29). Structure diagrams were drawn using PyMOL (Schrödinger, LLC) and Chimera (40). Phylogenetic analysis was performed and analyzed using MEGA. The RMSD values of ASFV dUTPase and swine dUTPase were calculated by the PDBeFold server (https://www.ebi.ac.uk/msd-srv/ssm/). The solvent-accessible surface areas and volumes of the substrate-binding pockets are calculated by CASTp (31). The surfaces of hydrophobicity distribution are completed with UCSF Chimera (40). The surfaces of electrostatic distribution are showed by using the PDB2PQR and APBS software (41, 42).

### Enzyme kinetics measurement

The hydrolysis of dUTP by a dUTPase is accompanied by proton release. The indicator phenol red can be protonated and deprotonated, with a maximum differential absorption at a wavelength of 559 nm. This is a common method used to measure dUTPase enzyme activity (43). The reaction system consisted of 1 mM Hepes (pH 7.5), 150 mM KCl, 40 μM phenol red, and 5 mM MgCl_2_. The protein concentration ranged from 3 to 50 nM, and the dUTP concentration ranged from 1 to 50 μM. A spectrophotometer and thermostatted cuvettes with a 10-mm path length were used at 25 °C to record the absorbance at 559  nm. The Michaelis–Menten equation was fit to the steady-state curves using GraphPad Prism. All measurements were carried out at least three times. Error bars represent standard deviations.

### Assessment of protein stability by thermofluor assay

The dUTPases were added to a capillary *via* a siphonage effect to track folding of the proteins by detecting their intrinsic fluorescence. The ratio of fluorescence signals changes with increasing temperature or concentration of a chemical denaturant, allowing for the determination of the melting temperature (*T*_*m*_) of a protein as a stability parameter. *T*_*m*_ was determined by nanodifferential scanning fluorimetry (nanoDSF) (Prometheus NT.48; Nanotemper Technologies, Munich, Germany) based on the ratio of tryptophan fluorescence at 350 nm/330 nm. Protein denaturation curves were determined in a temperature range between 20 and 95 °C with a slope of 7 °C·min^−1^. The *T*_*m*_ was calculated as the inflection point of the denaturation curve by first derivate analysis. All thermal stability experiments were repeated a minimum of three times, and the presented error bars indicate the standard deviations of these trials (44).

### Static light scattering assay

Light scattering measurements were performed using a DynaPro NanoStar instrument (Wyatt Technology Corporation). Before the measurements were taken, purified proteins in solution were centrifuged at 14,000 rpm at room temperature for 15 min. Protein concentrations were maintained at 5 mg/ml, and proteins were suspended in the same buffer used for the gel filtration experiments. The DYNAMIC software (v7.1.4) was used to obtain the normalized time autocorrelation function, polydispersity, and absolute molecular mass (45, 46).

### Inhibition kinetics measurement

And We synthesized two compounds (1 and 2) that were designed for *P. falciparum* dUTPase (47, 48). Compounds 1 and 2 were synthesized from RC ChemTec (Wuhan) company. A series of compound concentrations (0–100 μM final concentration at 5-fold serial dilution) in 100% DMSO were prepared. Then 5X (five times final concentration) compound solutions (5 mM Hepes [pH 7.5], 750 mM KCl, 200 μM phenol red, and 25 mM MgCl_2_) were prepared in assay buffer prior to assays. Four hundred microliters of 5X enzyme solution was distributed into wells, and 2 μl of dUTPase (1 mg/ml) and 2 μl of varying concentration of compounds (5X) were added and incubated for 30 min. The enzyme reaction was initiated by adding 100 μM of dUTP, and its activity was continuously detected for the production of ppi for 6 min. Enzyme activities of both ASFV dUTPase and swine dUTPase were monitored in the same way with varying concentrations of inhibitors and substrates (0–100 μM). The data were fit by using GraphPad Prism in order to determine the best fit inhibition mechanism and kinetic parameters for each compound.

### Surface plasmon resonance measurements

ASFV dUTPase was immobilized on a CM5 sensor chip using standard aminecoupling with running buffer PBST (10 mM Hepes, 150 mM NaCl, Tween-20 0.05%, 5% DMSO, pH 7.4) using a Biacore T200 instrument. ASFV dUTPase was immobilized to flow channels 2, and immobilization levels of flow channel 2 were ∼15,900 RU. Data were referenced with blank (enthanolamine) RU values. Compound solutions with a series of increasing concentrations (0–50 μM) were applied to channels 2 and 1 at a 30-μl/min flow rate. Response units were measured during the equilibration phase at each concentration, and the kinetic analysis was performed by SPR using the Biacore T200 evaluation software 2.0.

## Data availability

The coordinates and diffraction data for ligand-free aDUT, aDUT-dUPNPP-Mg^2+^, and aDUT-dUMP have been deposited in the Protein Data Bank with accession numbers 6LJO, 6LJ3, and 6LIS, respectively. The atomic coordinates and structure factors for sDUT-dUPNPP-Mg^2+^ have been deposited in the Protein Data Bank under accession number 6LJJ.

## Conflict of interest

The authors declare that they have no conflicts of interest with the contents of this article.
