# Supplementary material for: Structural comparisons of host and African swine fever virus dUTPases reveal new clues for inhibitor development
Source: J Biol Chem. 2020 Nov 23;296:100015. doi: 10.1074/jbc.RA120.014005 (PMC7948977; doi:10.1074/jbc.RA120.014005)
Supplement: Supplementary Figures and Tables [file mmc1.docx]

***Supplementary Materials for***

**Crystal structures of African swine fever virus dUTPase and swine dUTPase provide new clues for targeted inhibitor development**

**Rui Liang^1,2^, Gang Wang^1,2^, Ding Zhang^1,2^, Gang Ye^1,2^, Mengxia Li^1,2^, Yuejun Shi^1,2,3^, Jiale Shi^1,2^, Huanchun Chen^1,2^, Guiqing Peng^1,2 #^**

From the 1State Key Laboratory of Agricultural Microbiology, College of Veterinary Medicine, Huazhong Agricultural University; 2Key Laboratory of Preventive Veterinary Medicine in Hubei Province, The Cooperative Innovation Center for Sustainable Pig Production; 3College of Life Science and Technology, Huazhong Agricultural University.

Running Title: Crystal structure of ASFV dUTPase and swine dUTPase

# To whom correspondence should be addressed: Guiqing Peng: State Key Laboratory of Agricultural Microbiology, College of Veterinary Medicine, Huazhong Agricultural University, 1 Shi-zi-shan Street, Wuhan, 430070, China;

[penggq@mail.hzau.edu.cn](mailto:penggq@mail.hzau.edu.cn); Tel. +86 18071438015; Fax. +86 27 87280480.

**Keywords:** ASFV, swine, dUTPase, variable conformations, inhibitor design

**Supplementary Tables**

**Supplementary Figures**


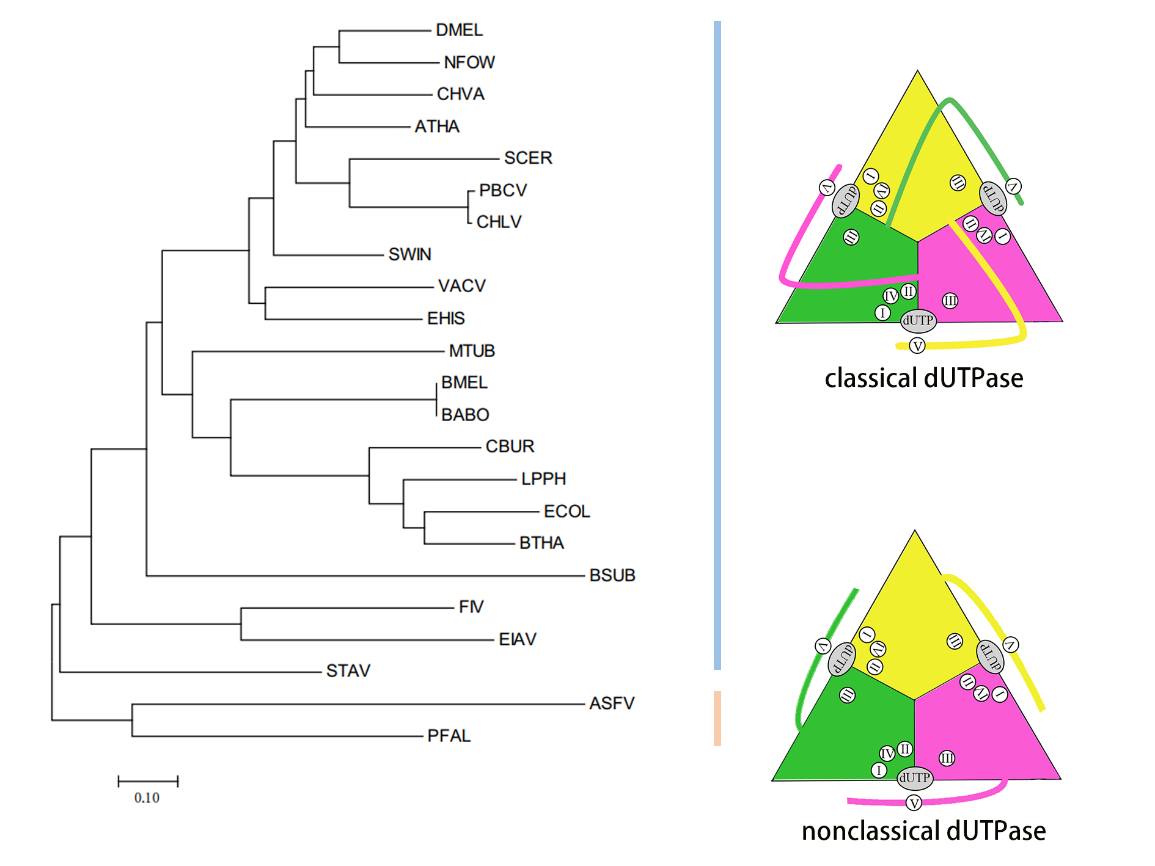


**Figure S1.** Phylogenetic analysis of dUTPase. DMEL, Drosophila melanogaster (PDB: 3ECY); NFOW, Naegleria fowleri (PDB: 5VJY); CHVA, Chlorella variabilis (PDB:3SO2); ATHA, Arabidopsis thaliana (PDB: 4OOP); SCER, Saccharomyces cerevisiae (PDB: 3HHQ); PBCV, Paramecium bursaria chlorella virus (PDB: 3CA9); CHLV, chlorella virus (PDB: 3C3I); SWIN, swine (PDB: 6LJJ); VACV, Vaccinia Virus (PDB: 2OKD); EHIS, Entamoeba histolytica (PDB: 3LQW); MTUB, M. tuberculosis (PDB: 1SLH); BMEL, Brucella melitensis (PDB: 3MDX); BABO, Brucella abortus (PDB: 3MBQ); CBUR, Coxiella burnetiid (PDB: 3TQZ); LPPH, Legionella pneumophila Philadelphia 1 (PDB: 6MAI); ECOL, Escherichia coli (PDB: 2HR6); BTHA, Burkholderia thailandensis (PDB: 4LHR); BSUB, Bacillus subtilis (PDB: 4APZ); FIV, feline immunodeficiency virus (PDB: 1F7K); EIAV, equine infectious anemia virus (PDB: 1DUN); STAV, Staphylococcus virus 11 (PDB: 4WRK); ASFV, African swine fever virus (PDB: 6LJ3); PFAL, Plasmodium falciparum (PDB: 2Y8C).


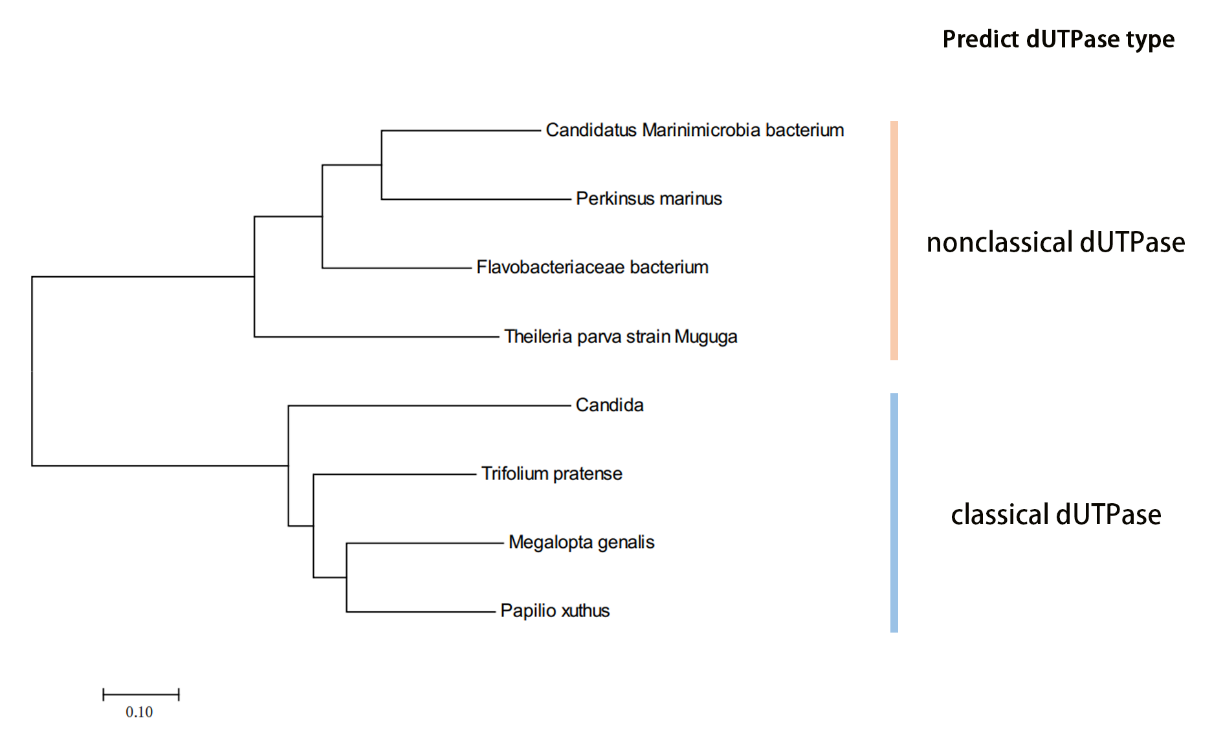


**Figure S2. Phylogenetic analysis of dUTPase.** Phylogenetic tree shows that primary sequences of dUTPases from different species are divided into two clusters.


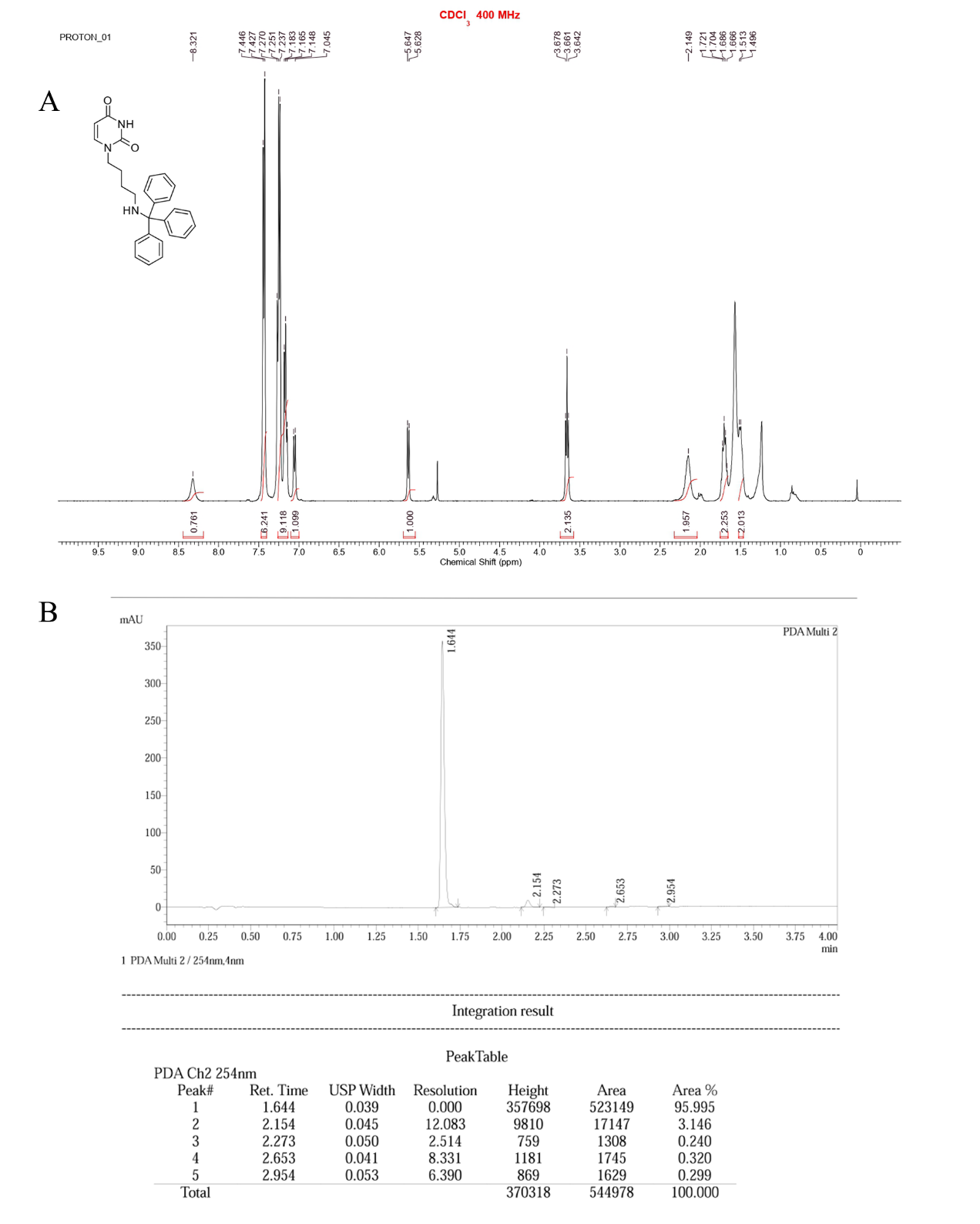


**Figure S3. NMR Spectra and HPLC analysis of compound 1. A.** Compound 1 structure was confirmed by NMR spectra. **B.** The HPLC analysis of compound 1. Compound purity was determined by HPLC to be ≥ 95%.


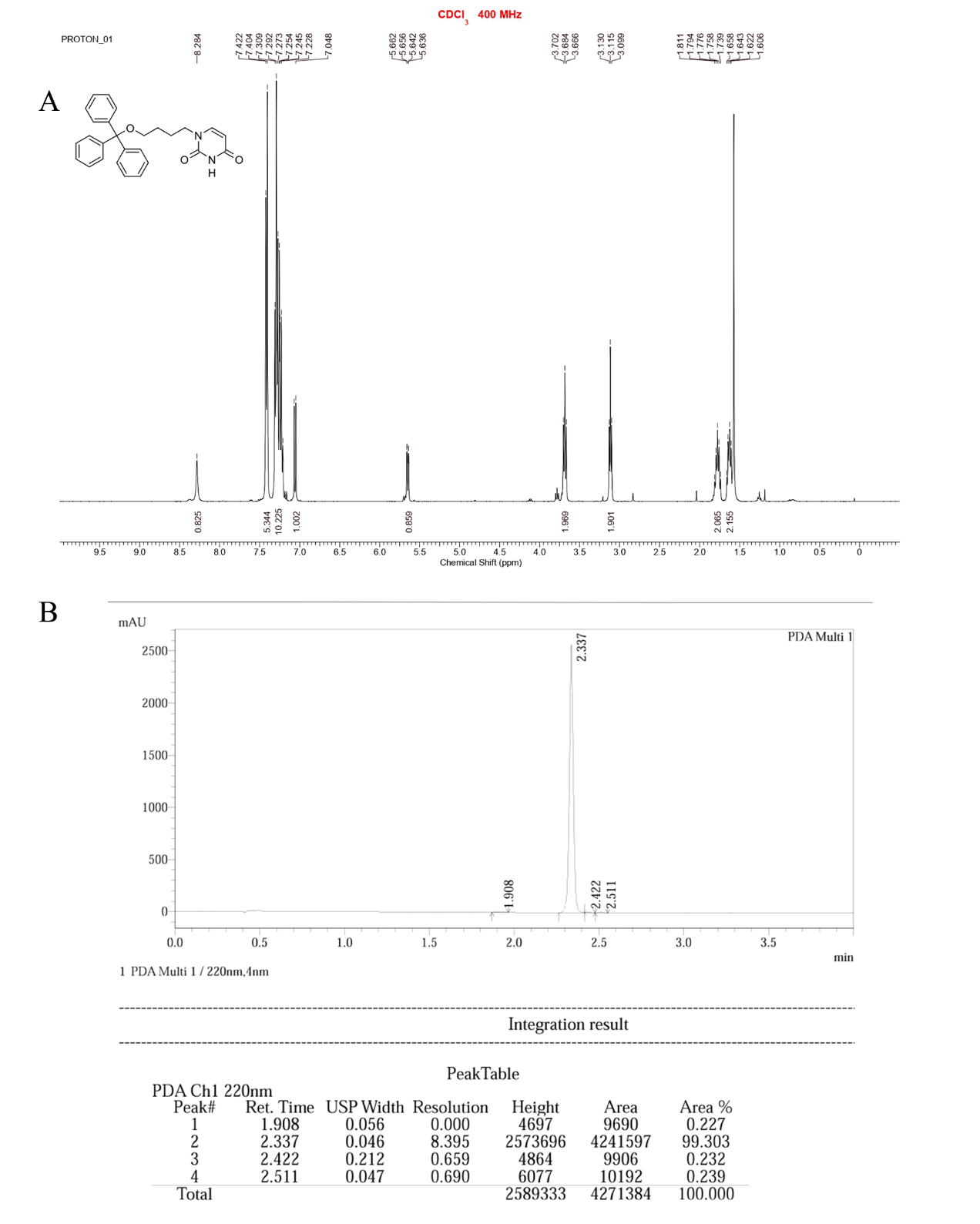


**Figure S4. NMR Spectra and HPLC analysis of compound 2. A.** Compound 2 structure was confirmed by NMR spectra. **B.** The HPLC analysis of compound 2. Compound purity was determined by HPLC to be ≥ 99%.

**Table S3. Comparison of the overall structures and active sites (RMSD, Å).**

| **overall** | **aDUT-dUMP** | **aDUT-dUPMPP-Mg^2+^** |
| --- | --- | --- |
| **aDUT** | 0.477 | 0.573 |
| **aDUT-dUMP** | \ | 0.362 |
| **aDUT-dUPMPP-Mg^2+^** | 0.362 | \ |
| **sDUT-dUPMPP-Mg^2+^** | 2.222 | 1.917 |

| **active sites** | **aDUT-dUMP** | **aDUT-dUPMPP-Mg^2+^** |
| --- | --- | --- |
| **aDUT** | 0.364 | 0.519 |
| **aDUT-dUMP** | \ | 0.347 |
| **aDUT-dUPMPP-Mg^2+^** | 0.347 | \ |
| **sDUT-dUPMPP-Mg^2+^** | 0.951 | 0.801 |
